# 558. Evaluation of BioFire FilmArray® Pneumonia PCR Multiplex Panel Testing in Routine Outpatient Surveillance Bronchoscopies After Lung Transplant

**DOI:** 10.1093/ofid/ofaf695.167

**Published:** 2026-01-11

**Authors:** Neeraja Swaminthan, Laura Frye, Kimberly Hanson, Hannah Imlay

**Affiliations:** University of Utah, Salt Lake City, Utah; University of Utah, Salt Lake City, Utah; -, Salt Lake City, Utah; University of Utah Health, Salt Lake City, UT

## Abstract

**Background:**

Lung transplant recipients (LTRs) undergo surveillance bronchoscopies (SB) at regular intervals post-transplant to detect rejection/infection early & guide immunosuppression. At our institution, SB with bronchoalveolar lavage (BAL) & transbronchial biopsy is performed until patients are rejection-free for 12 months. Microbiologic testing performed on BAL includes the BioFire FilmArray® Pneumonia panel (15 bacterial, 3 atypical, 8 viral targets, resistance genes), cultures, & additional targeted testing. While PCR panels provide faster results than culture, their role in routine SB is unclear. Moreover, outpatient multiplex PCR panels may not be reimbursed by patient’s insurance.

Bar graph showing the number of surveillance bronchoscopies (SBs) and the results of PCR and BAL bacterial culture testing.

**Figure:**
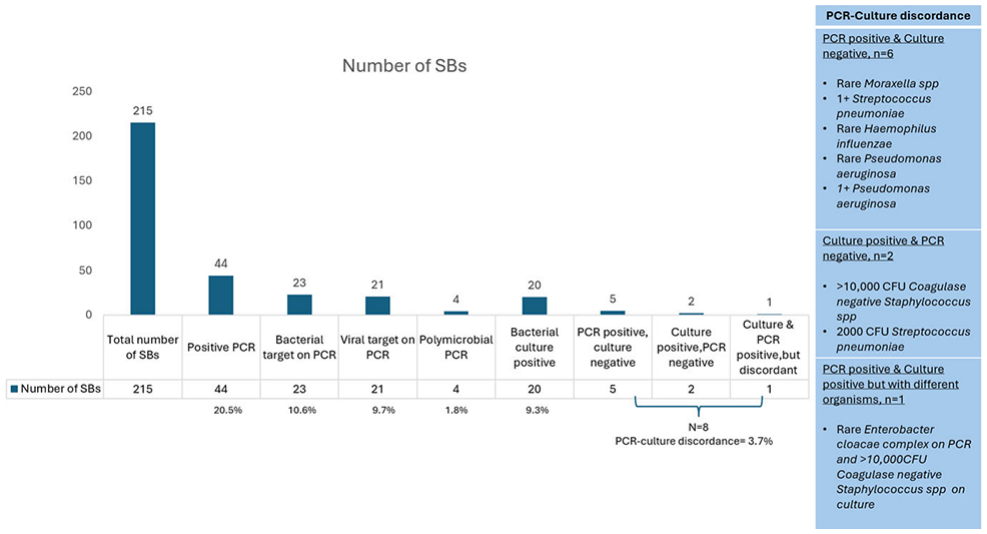

**Methods:**

We retrospectively analyzed all outpatient SBs in LTRs performed between October 2023 to April 2025. Patient demographics, results of testing, reported symptoms, & antibiotic prescriptions were recorded. We considered a culture as positive when specific organisms were identified & quantified. Concordance was defined as identical organisms or dominant organisms identified on both PCR panel & BAL bacterial culture. Instances where culture showed normal oral flora/no growth while PCR was negative were also considered concordant. Cohen’s kappa was used to assess PCR-culture agreement. BAL results for fungal/atypical pathogens were also recorded.

**Results:**

58 LTRs underwent 215 SBs. Mean age was 63; 70% were male. Most (96%) LTRs were double lung transplants. Median time from transplant to SB was 197 days (IQR 190.8). Only 7% of SBs occurred at a time when a patient had a clinical suspicion of possible LRTI. The PCR resulted with a positive target in 20.5% of BAL specimens,10.6% PCRs had a detected bacterial target, & 9.3% of bacterial cultures were positive (Figure 1). PCR-culture concordance was high (96%, kappa 0.79). 20 out of 23 BALs with positive bacterial targets on PCR were associated with an antibiotic prescription, including 3 cases with negative concomitant cultures. None of the PCRs identified resistance genes, but culture detected 2 instances of fluoroquinolone-resistant *P. aeruginosa*.

**Conclusion:**

Among LTRs undergoing SB, results identified from BioFire pneumonia panel were concordant with bacterial culture in most cases; the PCR panel adds cost without clear benefit.

**Disclosures:**

Hannah Imlay, MD, MS, Recursion pharmaceutical: Stocks/Bonds (Public Company)

